# Capillary density and caliber as assessed by optical coherence tomography angiography may be significant predictors of diabetic retinopathy severity

**DOI:** 10.1371/journal.pone.0262996

**Published:** 2022-01-26

**Authors:** Sam Kushner-Lenhoff, Kaitlin Kogachi, Melissa Mert, Zhongdi Chu, Anoush Shahidzadeh, Neal V. Palejwala, Jeremy Wolfe, Sujit Itty, Kimberly A. Drenser, Antonio Capone, Pravin U. Dugel, Andrew A. Moshfeghi, Hossein Ameri, Lauren P. Daskivich, Ruikang K. Wang, Amir H. Kashani

**Affiliations:** 1 Department of Ophthalmology, UW Medicine Eye Institute, University of Washington, Seattle, Washington, United States of America; 2 Department of Ophthalmology, Casey Eye Institute, Oregon Health & Science University, Portland, Oregon, United States of America; 3 Preventative Medicine, Southern California Clinical and Translational Science Institute, University of Southern California, Los Angeles, California, United States of America; 4 Department of Biomedical Therapeutics, University of Washington, Seattle, Washington, United States of America; 5 Department of Ophthalmology, USC Roski Eye Institute, University of Southern California, Los Angeles, California, United States of America; 6 Retinal Consultants of Arizona, Phoenix, Arizona, United States of America; 7 Associated Retinal Consultants, Oakland University of William Beaumont School of Medicine, Royal Oak, Michigan, United States of America; 8 Ophthalmic Services and Eye Health Programs, Los Angeles County Department of Health Services, Los Angeles, California, United States of America; 9 Department of Ophthalmology, Wilmer Eye Institute, Johns Hopkins University, Baltimore, Maryland, United States of America; 10 Department of Biomedical Engineering, Johns Hopkins School of Medicine, Baltimore, Maryland, United States of America; Massachusetts Eye & Ear Infirmary, Harvard Medical School, UNITED STATES

## Abstract

**Purpose:**

To validate retinal capillary density and caliber associations with diabetic retinopathy (DR) severity in different clinical settings.

**Methods:**

This cross-sectional study assessed retinal capillary density and caliber in the superficial retinal layer of 3-mm OCTA scans centered on the fovea. Images were collected from non-diabetic controls and subjects with mild or referable DR (defined DR worse than mild DR) between February 2016 and December 2019 at secondary and tertiary eye care centers. Vessel Skeleton Density (VSD), a measure of capillary density, and Vessel Diameter Index (VDI), a measure of vascular caliber, were calculated from these images. Discriminatory performance of VSD and VDI was evaluated using multivariable logistic regression models predicting DR severity with adjustments for sex, hypertension, and hyperlipidemia. Area under the curve (AUC) was estimated. Model performance was evaluated in two different cohorts.

**Results:**

This study included 594 eyes from 385 subjects. Cohort 1 was a training cohort of 509 eyes including 159 control, 155 mild non-proliferative DR (NPDR) and 195 referable DR eyes. Cohort 2 was a validation cohort consisting of 85 eyes including 16 mild NPDR and 69 referable DR eyes. In Cohort 1, addition of VSD and VDI to a model using only demographic data significantly improved the model’s AUC for discrimination of eyes with any DR severity from controls (0.91 [95% CI, 0.88–0.93] versus 0.80 [95% CI, 0.76–0.83], p < 0.001) and eyes with referable DR from mild NPDR (0.90 [95% CI, 0.86–0.93] versus 0.69 [95% CI, 0.64–0.75], p < 0.001). The transportability of this regression model was excellent when implemented in Cohort 2 for the referable DR versus mild NPDR comparison. The odds ratio of having any DR compared to control subjects, and referable DR compared to mild DR decreased by 15% (95% CI: 12–18%), and 13% (95% CI: 10–15%), respectively, for every 0.001 unit increase in VSD after adjusting for comorbidities.

**Conclusion:**

OCTA-derived capillary density has real world clinical value for rapidly assessing DR severity.

## Introduction

Diabetic retinopathy (DR) is one of the world’s leading causes of blindness [1] and its prevalence may be underestimated [2]. Pathologic findings such as microaneurysms, large-vessel dilation [3], and intraretinal hemorrhages are the defining features of DR because they are easily identifiable on fundus imaging and clinical exam. The Early Treatment of Diabetic Retinopathy Severity (ETDRS) is the most widely recognized staging system for DR classification but is primarily a research tool [4]. The current gold standard for clinical diagnosis and management of DR is the International Clinical Diabetic Retinopathy (ICDR) and Diabetic Macular Edema (DME) disease severity scales [5]. All of these scales rely on ophthalmoscopically visible changes in fundus appearance for staging. These macroscopic changes are widely believed to be the end result of many years of cumulative microscopic capillary pathology that are essentially undetectable on ophthalmoscopy [5]. Optical coherence tomography angiography (OCTA)—a commercially available, FDA approved, and noninvasive imaging technique—can capture these subtle capillary changes [6–14]. As a result, it may be useful in evaluating DR severity where access to ophthalmologists is limited and in early stages of disease when invasive fluorescein angiography is neither available nor indicated.

Vessel skeletal density (VSD) is a dimensionless measure of capillary density representing the total linear length of vessels in an OCTA image normalized to the size of the OCTA image [7, 15, 16]. This characteristic is known to correlate with DR severity and other systemic risk factors [7, 11, 13, 17]. Vessel caliber as reported by vessel diameter index (VDI) is a related but distinct measure of vessel morphology that has been similarly identified as a useful OCTA metric in DR [7]. VDI is the average width, in pixels, of the vessels in an OCTA image.

In this study, we determine the robustness of OCTA-derived retinal capillary density and caliber in discriminating DR severity. We then evaluate the transportability, accuracy, and utility of these metrics in two distinct clinical settings [18, 19]. In using multiple sites and comparing these metrics to demographic risk factors for diabetic retinopathy [20], we provide an initial assessment of the clinical utility of these OCTA measures.

## Materials and methods

### Study design

This is a multicenter, cross-sectional analysis of prospectively collected subject data that was collected from spatially and temporally distinct cohorts. Cohort 1 included subjects from three geographically separate tertiary-care retina referral centers—the USC Roski Eye Institute (Los Angeles, CA), Retinal Consultants of Arizona (Phoenix, AZ) and Associated Retinal Consultants (Royal Oak, MI). Cohort 2 included subjects from a separate DR teleretinal screening center located at Los Angeles County + University of Southern California (LAC+USC) Medical Center (Los Angeles, CA). Approval was obtained from the University of Southern California Institutional Review Board (IRB) and Western IRB. The study adhered to the tenets of the Declaration of Helsinki. Subjects consented to have their data available for research purposes and this manuscript is based on secondary analyses of prospectively collected data.

For Cohort 1, inclusion criteria for non-diabetic controls was availability of ophthalmological exam results and OCTA images at the time of clinical exam and no self-reported history of diabetes mellitus (DM). These were non-diabetic subjects where the examining ophthalmologist ruled out any significant ocular pathology. OCTA imaging is standard-of-care at these retina specialist clinics. Diabetic subjects were included if they had a known diagnosis of any DM, ophthalmological exam results which showed at least mild DR, and OCTA images at the time of clinical examination. Subject data was collected from February 2016 and June 2019.

For Cohort 2, non-diabetic controls were not available because only diabetic subjects were referred for DR screening in the LAC+USC Teleretinal DR Screening clinic [21]. Subjects with diabetes were included in Cohort 2 if they had a conclusive DR diagnosis (with an official result in the teleretinal medical record) and simultaneous OCTA imaging. Data was collected between April and December 2019.

Exclusion criteria for all subjects were use of any intraocular pressure lowering drops, any significant media opacity, and any other ophthalmic diagnosis except dry eye, vitreous syneresis, posterior vitreous detachment, asymptomatic epiretinal membrane without retinal distortion, peripheral retinal tear not requiring incisional surgery, and trauma without any notable sequelae.

DR in subjects from Cohort 1 was classified based on a comprehensive ophthalmologic evaluation including dilated fundus examination performed by a board-certified and fellowship-trained retina specialist. DR categories were mild NPDR, moderate NPDR, severe NPDR and PDR based on the criteria set by the International Clinical DR and DME severity scales [5]. For subjects in Cohort 2, classification of DR was made using 3 nonmydriatic 45-degree digital fundus photographs which has significant agreement with 7-field ETDRS DR staging [22, 23]. DR staging was performed by an optometrist specifically trained and certified for teleretinal DR screening at the Los Angeles County screening clinic. This expert’s evaluation was then independently reviewed and adjudicated by one of our board certified and fellowship trained retina specialists. For all subjects, DME was assessed by manual review of all OCT B-scans. Demographic and clinical history including age, sex, and diagnoses of hyperlipidemia, hypertension, and diabetes were obtained from medical records in both cohorts.

OCTA at all sites was performed using a commercially available CIRRUS AngioPlex 5000 (Carl Zeiss Meditec, Dublin, CA, USA). 3mm x 3mm images consisting of 245 horizontal B-scans and 245 individual A-scans per B-scan were obtained. OCTA images were included if they had a signal strength > 7, were centered on the fovea, lacked significant hypo- or hyperreflective artifacts on *en face* structural images, and had fewer than 10 motion artifacts. If both eyes of a subject met the inclusion criteria, a highest quality image (fewest number of imaging artifacts) was determined as well.

The superficial retinal layer (SRL) was the layer analyzed in all eyes to minimize the confounding impact of projection artifacts, projection artifact removal software, or layer segmentation. The commercially available Cirrus AngioPlex automated segmentation algorithm defined the SRL from the internal limiting membrane to the outer boundary of the inner plexiform layer. All OCT B-scan segmentation lines were reviewed for SRL segmentation errors and none of the images included in the final analysis required manual resegmentation.

A previously validated, semi-automated, custom MATLAB (MathWorks Inc., Natlick, MA) algorithm was applied to a grayscale bitmap image of the SRL exported from the AngioPlex machine to calculate the VSD and VDI for each image [6–8, 24–26]. VSD is a measure of vascular length and is computed by skeletonizing all vessel segments to a width of one pixel, counting the number of pixels that represents all skeletonized vessels and dividing that by the total number of pixels in the image [7, 15]. VDI is derived by taking the number of pixels showing blood flow in a binarized OCTA image and dividing that by the number of pixels showing blood flow in a skeletonized OCTA image [7, 15].

### Statistical analysis

All statistical analysis and regression models were performed using SAS Version 9.4 (Cary, North Carolina). A post-hoc analysis of study power was performed. We found that our sample size provides more than 80% power for detecting significant differences between the control and any DR groups and the mild DR and referable DR groups for VSD or VDI.

A mixed effects multinomial logistic regression model was developed using subjects from Cohort 1 as the training cohort and modeling disease severity as the dependent nominal variable with 3 levels including control, mild, and referable DR (a previously defined group [27] which included moderate/severe NPDR, and PDR). Routinely collected clinical and demographic data shown to be potential predictors of DR were considered for model inclusion with VSD and VDI: these included age, sex, hypertension, and hyperlipidemia. Variables significantly associated with the DR severity outcome in a univariable model were selected for a multivariable model using a backward and stepwise elimination method until a parsimonious model with all variables statistically significant at p < 0.05 via the Satterthwaite denominator degree-of-freedom method was obtained. During this process, age was removed from the model, so that the complete model included VSD, VDI, sex, hypertension, and hyperlipidemia. In other words, age was not a significant confounder for our models and thus age was not retained as a covariate. Other work has previously failed to find an association between age and severity of retinopathy [28]. All models were adjusted for the random effects of eye-nesting within subject and ophthalmic center. The Newton-Raphson with ridging optimization technique was utilized to help with the convergence of the procedures. All models we subsequently describe show acceptable goodness-of-fit with the Hosmer-Lemeshow test p > 0.05, indicating our models are well-calibrated.

The discriminatory performance of the final multivariable model in determining any DR versus controls and referable DR versus mild DR was assessed by generating receiver operating characteristic (ROC) curves and calculating the area under the curve (AUC).

For each classification of interest, the AUC from models including VSD, VDI, or both VSD and VDI was compared nonparametrically to a general covariate model called “Model 1” that included only sex, hypertension, and hyperlipidemia in order to determine the incremental discriminatory contribution of OCTA metrics [29]. The p-value for the null hypothesis that the difference in the AUCs = 0 was generated by estimating a covariance matrix using the method of structural components and computing the test statistic having an asymptotically chi-square distribution [29].

Each constrained comparison of interest was further internally validated for the complete prediction model using ten-fold cross validation. This involved splitting the data into ten equally sized groups, estimating the model coefficients on 9 of the 10 groups, and then evaluating its accuracy in the group held out. The AUC was averaged over the 10 validation sets and reported as the evaluation metric. The prediction model derived from Cohort 1 was then applied to data from Cohort 2 which served as a validation data set. Model performance for the referable DR versus mild DR comparison in Cohort 2 was similarly quantified by calculating the AUC; incremental contributions of VSD and VDI were assessed as described for Cohort 1.

Using one highest quality single image for each subject in both cohorts, ROC-based thresholds were used to estimate the optimal values for both VSD and VDI in discriminating any DR versus control and referable DR versus mild DR. The threshold giving the highest correct classification rate was chosen as the most appropriate summary measure.

## Results

A total of 594 eyes (385 subjects) were included in both cohorts (Table 1). In Cohort 1, all subject characteristics (age, sex, hyperlipidemia, hypertension, and DME) were found to differ significantly between groups and were considered potential predictors of DR, except for DME because it perfectly predicted DR.

**Table 1:** Demographics.

	DR Severity in Cohort 1			p-value	DR Severity in Cohort 2		p-value
	Control	Mild	Referable		Mild	Referable	
N eyes	159	155	195	-	16	69	-
N subjects ^c^	102	100	129	-	12	42	-
**Characteristics of subjects**:							
Mean Age, years (SD)	51.0 (17.9)	57.3 (13.4)	57.3 (12.5)	0.002 ^b^	50.5 (15.0)	54.0 (10.5)	.35 ^b^
Age range, years	21–86	23–80	29–91	-	28–68	23–86	-
Female sex, No. (%)	61 (59.8%)	42 (42.0%)	63 (48.8%)	0.04 ^a^	6 (50.0%)	17 (40.5%)	0.74 ^a^
Hyperlipidemia, No. (%)	9 (8.8%)	37 (37.0%)	58 (45.0%)	<0.001 ^a^	5 (41.7%)	18 (42.9%)	1.00 ^a^
Hypertension, No. (%)	18 (17.6%)	53 (53.0%)	91 (70.5%)	<0.001 ^a^	7 (58.3%)	26 (61.9%)	1.00 ^a^
DME, No. (%)	0 (0.0%)	9 (9.0%)	65 (50.4%)	<0.001 ^a^	2 (16.7%)	18 (42.9%)	0.17 ^a^

^a^Chi-squared test or Fisher’s exact test, as appropriate.

^b^One-way analysis of variance.

^c^Subjects with eyes of varying DR severity were counted as part of their least severely diseased eye.

**Fig 1:**
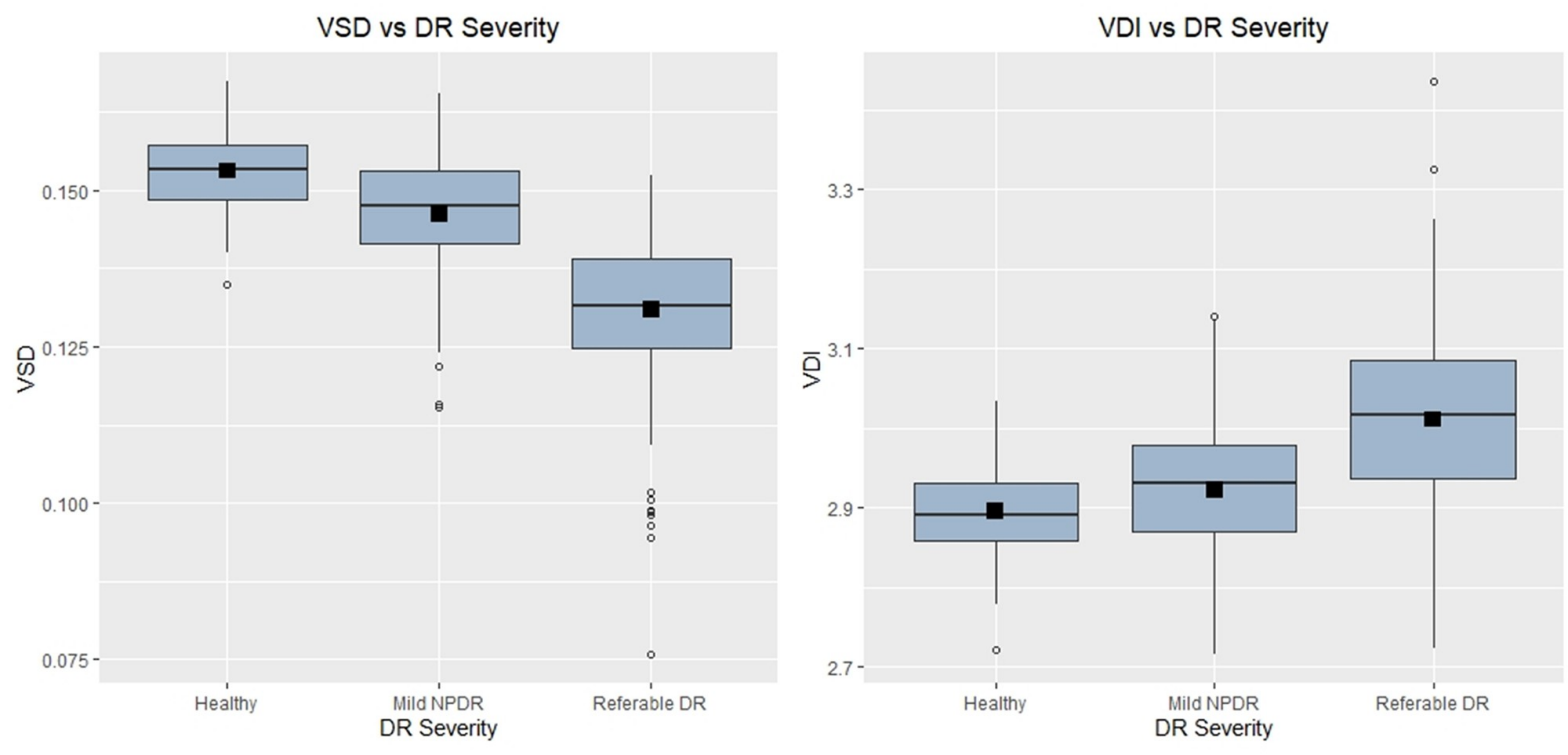
Box and whisker plots of the VSD and VDI distribution in the tertiary referral centers (Cohort 1). Start and end of boxes represent 25^th^ and 75^th^ quartiles respectively. Vertical lines extend to the smallest and largest values no further than 1.5 times the interquartile range. The middle horizontal line represents the median and the black square box represents the mean. Outliers are plotted as circles.

The VSD and VDI were computed for each subject and the distribution of the data in Cohort 1 is shown in Fig 1. The VSD, mean [SD], for the control group, mild DR group and referable DR group in Cohort 1 was 0.153 [0.006], 0.146 [0.010], and 0.131 [0.012], respectively, while the VDI for each of these groups was 2.90 [0.05], 2.92 [0.08], and 3.01 [0.11], respectively. In Cohort 2, the VSD, mean [SD], for the mild and referable DR categories was 0.149 [0.007] and 0.135 [0.014], respectively, while the VDI for each of these groups was 2.93 [0.06] and 3.00 [0.09], respectively.

The odds ratios for changes in individual components on which the complete multivariate model was built are shown in Table 2. After refitting the model to produce constrained comparisons of interest, the odds ratio of having any DR compared to control subjects and referable DR compared to mild DR decreased by 15% (95% CI: 12–18%), and 13% (95% CI: 10–15%), respectively for every 0.001 unit increase in VSD. The odds of having any DR compared to control subjects and referable DR compared to mild DR increased by 10% (95% CI: 7–13%) in both cases for every 0.01 unit increase in VDI.

**Table 2:** Complete multivariable model odds ratios.

Predictor	OR (95% CI)		p-value
	Mild versus Control	Referable versus Control	
**VSD** ^1^	0.89 (0.85, 0.93)	0.78 (0.74, 0.82)	<0.001
**VDI** ^1^	1.05 (1.01,1.09)	1.13 (1.08,1.18)	<0.001
**Male**	2.63 (1.52, 4.55)	1.32 (0.66, 2.63)	<0.001
**Hyperlipidemia**	3.54 (1.61,7.80)	2.40 (0.96,5.99)	0.006
**Hypertension**	3.29 (1.76,6.18)	5.81 (2.69,12.54)	<0.001

^1^ORs and their 95% CIs are reported per 0.001 unit increase in VSD and 0.01 unit increase in VDI.

### Multivariate model performance in discriminating between any DR severity versus control and referable DR versus mild NPDR

**Fig 2:**
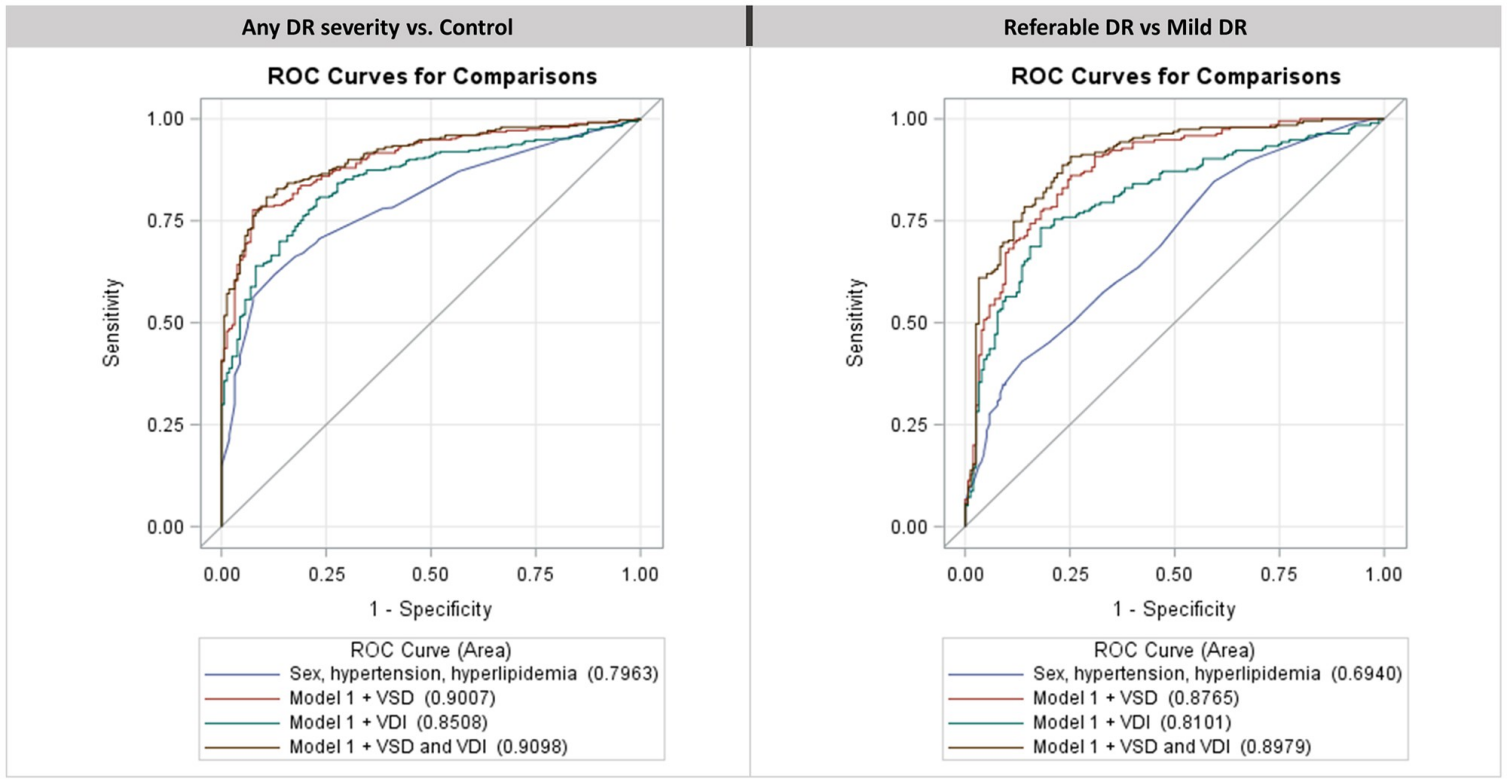
Receiver operator characteristic (ROC) curves. ROC curves for the constrained comparisons of any DR severity versus control and referable DR versus mild non-proliferative DR in Cohort 1. Additional lines are drawn for the sequential addition of VSD and VDI as parameters to the baseline model (labeled Model 1 and drawn in blue) containing sex, hypertension, and hyperlipidemia. A red line is drawn for a model with the parameters for Model 1 with VSD (red), a green line is drawn for a model with the parameters of Model 1 with VDI (green), and a brown line is drawn for the complete model containing the parameters of Model 1 with VSD and VDI (brown). Area under the curve for each ROC curve is listed in parenthesis next to each model in the legend.

Fig 2 shows the ROC curves for classifications of any DR versus control and referable DR versus mild DR in Cohort 1. The AUCs for Model 1 classifying any DR versus control and referable DR versus mild DR as seen in Table 3 were 0.80 (95% CI: 0.76–0.84) and 0.69 (95% CI: 0.64–0.75), respectively. The addition of VSD significantly (p < 0.001) increased Model 1’s AUC by 0.10 to 0.90 (95% CI: 0.87–0.93) and by 0.19 to 0.88 (95% CI: 0.84–0.91) for any DR versus control and referable DR versus mild DR, respectively. The addition of VDI to Model 1 increased the AUC by 0.05 to 0.85 (95% CI: 0.82–0.88) and by 0.12 to 0.81 (95% CI: 0.76–0.86) respectively, for any DR versus control and referable DR versus mild DR. A comprehensive model with VSD, VDI, sex, hypertension, and hyperlipidemia was significantly (p ≤ 0.02 for every case) superior in its discriminatory ability compared to any other combination of comorbid factors with VSD or VDI, with an AUC of 0.91 (95% CI: 0.88–0.93) and 0.90 (95% CI: 0.86–0.93) for any DR versus control and referable DR versus mild DR, respectively. Model AUCs along with sensitivity and specificity are shown in Table 3.

**Table 3:** Model evaluation.

DR Comparison	Model	Internal Validation AUC (95% CI)	AUC (95% CI)	p-value	Sensitivity (95% CI)	Specificity (95% CI)
**Performance of Cohort 1 Model**						
*Any DR severity (mild and referable DR) versus Control*	Model 1^a^ + VSD + VDI	0.90 (0.87–0.93)	0.91 (0.88–0.93)	<0.001 ^b^	0.89 (0.85–0.92)	0.69 (0.61–0.76)
	Model 1^a^		0.80 (0.76–0.84)			
*Referable DR versus Mild DR*	Model 1^a^ + VSD + VDI	0.87 (0.83–0.91)	0.90 (0.86–0.93)	<0.001 ^b^	0.89 (0.83–0.93)	0.66 (0.58–0.73)
	Model 1^a^		0.69 (0.64–0.75)			
**Performance of Cohort 1 Model Applied to Cohort 2**						
*Referable DR versus Mild DR*	Model 1^a^ + VSD + VDI		0.82 (0.70-.93)	<0.001 ^b^	0.75	0.68
	Model 1^a^		0.46 (0.32-.61)			

^a^Model 1 represents the multivariate model consisting of the subject’s sex, hypertensive status, and hyperlipidemia status.

^b^p-value for ROC contrast with Model 1.

#### Internal validation

For the complete model predicting any DR versus control and referable DR versus mild DR from Cohort 1, internal validation from the 10-fold cross validation reinforced strong discriminatory ability, with a mean AUC across all repetitions of cross-validation of 0.90 (95%: 0.87–0.93) and 0.87 (95%: 0.83–0.91) respectively.

#### External validation

Table 3 also summarizes the results of the external validation and transportability of the model developed in Cohort 1 when applied to data from Cohort 2. Notably, VSD and VDI each individually significantly contribute to the predictive performance of the model in Cohort 2 as they did in Cohort 1. Compared to the Model 1 AUC of 0.46 (95% CI: 0.32–0.61) in this population, VSD increased the model AUC by 0.35 to 0.81 (95% CI: 0.69–0.93) and VDI increased the model AUC by 0.22 to 0.68 (95% CI: 0.54–0.82) in Cohort 2. The complete model with VSD and VDI was significantly better at discriminating referable DR from mild DR subjects (AUC: 0.82, 95% CI: 0.70–0.93) than Model 1 although it was not significantly different than Model 1 with VSD (p = 0.79).

#### Optimal VSD and VDI cutoff points

After combining both cohorts, a VSD cutoff of 0.153 (correct classification rate of 0.84) and 0.143 (correct classification rate of 0.79) best discriminated subjects with any DR from control and subjects with referable DR from mild DR, respectively. Similarly, the VDI cut points were 2.91 (correct classification rate of 0.82) and 2.97 (correct classification rate of 0.72) for the same comparisons, respectively.

## Discussion

Clinical classification of DR is a critical step in the management of DR but it is also a laborious process for patients and physicians. Clinically feasible screening methods can play an important role in improving DR classification and clinical care. This is particularly true in the early stages of DR when ophthalmoscopic findings are subtle and clinical examination is therefore of low yield. In this study, we demonstrate that vessel skeleton density (VSD) significantly improves discrimination of clinically important DR categories in comparison to age, sex, and medical comorbidities alone among a population of subjects evaluated for DR by a retina specialist. In addition, the same finding holds true when VSD is applied in a cohort of patients referred for evaluation of DR via a teleretinal screening clinic using fundus photography. These findings suggest that OCTA-derived metrics can significantly improve or complement staging of DR in real-world clinical settings.

This additional discriminatory ability beyond demographic factors is likely due to the near histologic resolution of OCTA [30] that allows unprecedented detection of flow impairment. As our models show, this provides a significant advantage that can be leveraged in the clinical setting to improve disease classification even without knowledge about the funduscopic appearance of the retina or invasive laboratory tests such as HgA1c. Moreover, the earliest signs of DR, such as dot-and-blot hemorrhages and microaneurysms, can appear and disappear over time thereby eluding ophthalmoscopic detection. Even when present, these lesions can be subtle and difficult to detect reliably in a busy clinical setting. In contrast, OCTA-derived measures of capillary density provide an optical biopsy of retinal capillary perfusion that reflects the accumulating capillary pathology from DM rather than the transient ophthalmoscopic findings characteristic of early DR [31].

Our analysis was performed on subject data generated by 9 board certified and fellowship-trained retina specialists located in three geographically separate tertiary care retina referral centers. Therefore, we believe that the data used in our modeling represents a reasonable sample of clinical DR classification as it occurs in the United States. Ten-fold internal cross-validation of our results further confirmed our findings. The training data of Cohort 1 was derived from subjects that come from relatively affluent and insured patient populations. In order to demonstrate that our model is applicable in other settings, we used it to categorize images from a cohort of primarily Latino, low-income, and under-insured patients from a teleretinal DR screening clinic in Los Angeles County, Cohort 2. The test cohort in this case was created to validate the conclusions of Cohort 1 in a rigorous way by taking advantage of the multicenter nature of this study [32]. The significant incremental improvement in discriminating referable DR from mild DR in this validation cohort demonstrates the robust and reliable nature of OCTA-derived capillary density. Perhaps the most important finding of this study was the validation of the complete model generated from Cohort 1 in the external validation cohort in the most clinically relevant comparison of referable DR versus mild DR.

OCTA-derived capillary density appears to be particularly sensitive to DR with a decrease in the odds of any DR versus control and referable DR versus mild DR ranging from 13–15% for every 0.001 increase in VSD. Since VSD values typically decrease in subjects with increasing severity of DR it can be more intuitive to consider the inverse [7]. For every 0.001 unit decrease in VSD, the odds of having any DR compared to control subjects and referable DR compared to mild DR increases by 18% (95% CI: 14–22%) and 15% (95% CI: 11–18%), respectively. The range of capillary density in the Cohort 1 dataset spanned VSD values of 0.076 to 0.167. This is orders of magnitude larger than the incrementally significant change in VSD of 0.001 which means that large VSD changes suggest dramatically increased risk. Our analysis also suggests the addition of other OCTA-derived metrics (e.g. VDI) may be useful as well [33]. In the future, the accuracy of the categorization of DR may be further increased by using techniques such as image averaging [34].

### Limitations

One possible limitation of this study is that we did not analyze the deep retinal layer (DRL) though this decision was made for several reasons [17, 35–38]. Durbin et al. have reported that the AUC of retinal capillary density in the SRL was the best for detecting DR [11]. SRL vessel density changes may also be more reliably detectable than those in the DRL [39–41]. SRL analysis also does not require projection artifact removal which is available but not clearly validated against a meaningful ground truth [14, 36, 42, 43]. Reproducibility and repeatability has been shown in the SRL [44] as well. Though axial length calibration may also affect the VSD and VDI measurements [45], this information is not readily available at a diabetic retinopathy screening center which comprised our validation cohort.

The nature of this study as a secondary analysis created additional limitations. This prevents us from claiming that this is a predictive model as patients were not prospectively categorized before classification by the ophthalmologist. Furthermore, the model does not incorporate certain patient demographics including those with diabetes without DR, and thus the any DR versus control comparison could not be validated in our external cohort. All of these concerns could be further addressed by a prospective study.

## Conclusions

VSD and VDI calculated from OCTA images of the SRL provide additional information in discriminating between subjects with DR and healthy individuals and between subjects with referable DR and mild DR beyond that of common medical historical covariates: age, sex, hyperlipidemia, and hypertension. Our model’s high performance in differentiating referable DR from mild DR was transportable from a tertiary care cohort to a teleretinal screening cohort. In each of these cases, VSD showed more robustness across these comparisons than VDI in the DR subject population.
